# A Novel Approach for Repetitive Dislocation of Transvenous Left Ventricular Leads During Cardiac Resynchronization Therapy Implantation by the Loop Technique

**DOI:** 10.3389/fcvm.2022.836514

**Published:** 2022-06-21

**Authors:** Hao-Yu Wu, Shang-Jian Li, Zheng Yang, Hai-Chao Chen, Peng-Hua You, Gong Cheng

**Affiliations:** Department of Cardiology, Shaanxi Provincial People’s Hospital, Xi’an, China

**Keywords:** cardiac resynchronization therapy, heart failure, left ventricular lead dislocation, loop technique, novel technique

## Abstract

Cardiac resynchronization therapy (CRT) for heart failure requires transvenous insertion of a left ventricular pacing lead through the coronary sinus. However, repeated intraoperative dislocations often occur. Therefore, we describe a novel technique that uses the loop technique to treat patients with repeated intraoperative dislocations during transvenous left ventricular lead implantation to stabilize the lead in its final position. In five patients with repeated intraoperative dislocation during transvenous left ventricular lead implantation, the loop technique was successfully used to stabilize the lead in its final position. The pacing and sensing parameters were satisfactory in all patients at implantation and 12 months post-operatively. Compared with the pre-operative values, the 12-month post-operative values for the left ventricular ejection fraction were significantly increased and the left ventricular end systolic dimension and left ventricular end diastolic dimension were significantly decreased (*P* < 0.05). The left ventricular ejection fraction of these 5 patients increased by more than 15%. CRT significantly improved the left ventricular structure and function of these 5 patients. During the 1-, 3-, 6-, and 12-month follow-ups, no left ventricular lead dislocations were observed. This loop technique is safe and effective and can be considered for repeated intraoperative dislocation during transvenous left ventricular lead implantation through the coronary sinus of a CRT device.

## Introduction

The prevalence of heart failure is gradually increasing, and it has a high mortality rate (1). The treatment of heart failure has evolved in the past few years due to new evidence supporting optimal medical therapies and prognosis has improved (2, 3). However, heart failure continues to have a significant adverse impact on individual patients and the wider society. Cardiac resynchronization therapy (CRT) revolutionized the treatment for a well-defined subgroup of heart failure to reduce mortality and hospitalization by restoring the synchronicity of the intraventricular,

interventricular, and atrioventricular contractions (4–6). The first-line approach involves implanting pacing leads into the left ventricles through the coronary sinus. However, this technique remains challenging, and many complications, such as transvenous left ventricular lead failure, perforation and dissection, have been reported (7). Initially, transvenous left ventricular lead implantation had a failure rate of 10–15% at implantation and during short-term follow-up. Although this high failure rate has been remarkably improved in recent years, dislocation of the left ventricular lead through the coronary sinus still occurs in approximately 5–10% of cases (8–10). Therefore, to achieve CRT for these patients, alternative approaches are necessary.

In this study, we describe a novel and effective technique using the loop technique for repeated intraoperative dislocations during transvenous left ventricular lead implantation of a CRT device.

## Materials and Methods

### Patient Enrollment

Between December 2017 and March 2020, patients with advanced heart failure and widened QRS complex received CRT. All symptomatic patients had left ventricular ejection fraction ≤35% in NYHA Class III or IV with a prolonged QRS duration > 130 ms despite optimal medical therapy. CRT implantation criteria met the guidelines (11). All patients provided informed consent before the operation.

### Cardiac Resynchronization Therapy Implant Procedure

Patients were under local anesthesia and conscious sedation during the operation. The left subclavian vein was used to introduce separate guidewires for the right atrial, right ventricular, and left ventricular leads. A balloon occlusive venogram was performed after coronary sinus cannulation in every patient to select the target vein (lateral or posterolateral vein). Whenever repeated left ventricular lead dislocations (≥2 dislocations) were encountered, by protocol at least one other lead configuration (usually from a different manufacturer) was tried. Finally, an attempt was made to position the left ventricular lead by using the loop technique according to the protocol.

### Loop Technique Procedure

**FIGURE 1:**
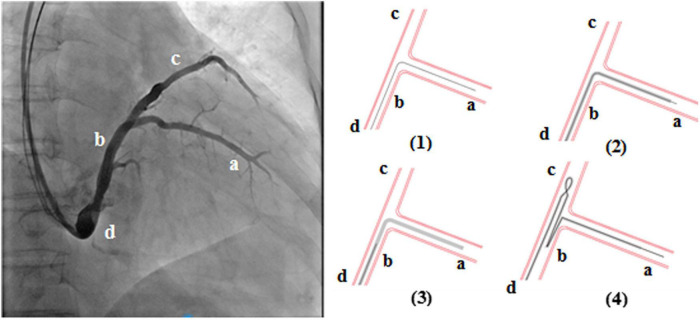
The loop technique for the left ventricular lead was achieved in a stepwise manner.

The loop technique procedure for the left ventricular lead was achieved in the following stepwise manner (Figure 1): (1) the guidewire (PTCA wire) was sent to the target vessel “a” through the coronary sinus; (2) the advancement of the left ventricular lead tip to the appropriate position of the target vessel; (3) the left ventricular lead tip position was kept unchanged, and the guidewire tip retreated to position “b”; and (4) the advancement of the guidewire tip in the left ventricular lead to position “c,” and the left ventricular lead body forms a loop structure.

If acceptable parameters were obtained in repeated measurements, the guidewire was removed, and the lead was sutured. The position of the left ventricular lead in the RAO 30° and LAO 60° projections was recorded. Finally, the subcutaneous tissue was adjusted, and the skin was closed.

### Follow-Up

Follow-up parameters were measured pre-discharge and 1, 3, 6, and 12 months post-implantation. In addition, at the 12-month follow-up, chest X-ray images were repeated in the same projection to verify the left ventricular lead position.

### Statistical Analyses

All statistical analyses were performed with SPSS version 20 (SPSS Inc., IBM Corp, Chicago, Illinois, United States). Continuous variables were presented as the mean ± SD, and the pre-operative and post-operative differences were compared using Student’s *t* test. A *P* value of <0.05 was considered statistically significant.

## Results

### Patient Demographics

**FIGURE 2:**
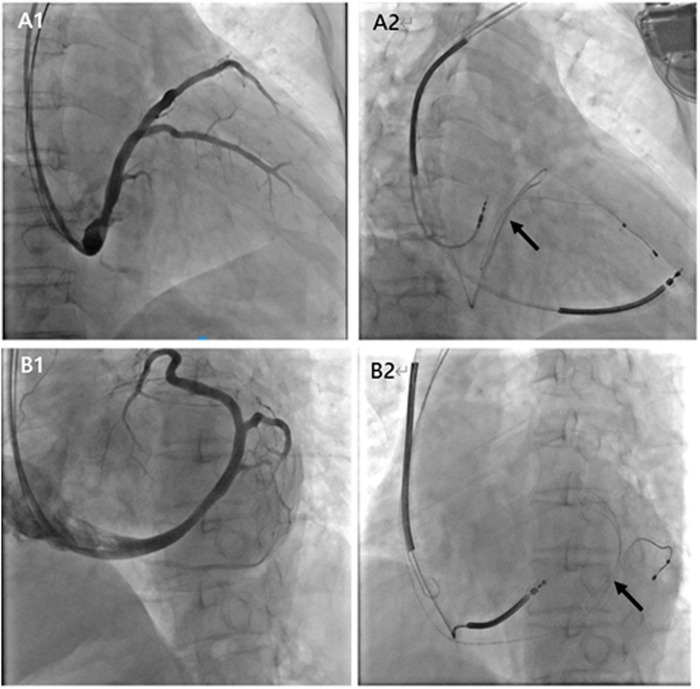
Coronary sinus angiography from RAO 30° **(A1)** and LAO 60° **(B1)** projections. Final position of the left ventricular lead by the loop technique from different projections **(A2,B2)**. The black arrows show the loop in the location of the coronary vessel.

In the 64 consecutive patients selected for CRT, 5 patients (7.81%) had repeated intraoperative left ventricular lead dislocation, including 2 males and 3 females with a mean age of 60 years old (from 55 to 65 years old). Four patients were diagnosed with dilated cardiomyopathy, and 1 patient was diagnosed with coronary heart disease (Table 1). The pre-operative left ventricular ejection fraction (LVEF), left ventricular end systolic dimension (LVESD), and left ventricular end diastolic dimension (LVEDD) were 25.6 ± 3.5%, 69.6 ± 9.6 mm, and 75.8 ± 9.8 mm, respectively. The loop technique was successfully used to stabilize the left ventricular lead in its final position in 5 patients. Figure 2 shows the final position of the left ventricular lead from different projections.

**TABLE 1:** Patient characteristics before operation.

Patient no.	Age (y)	Sex (M/F)	LVEF (%)	LVESD (mm)	LVEDD (mm)	NYHA pre-operative	Underlying heart disease
1	65	F	28	62	68	III	DCM
2	55	M	26	63	71	IV	DCM
3	60	M	25	85	92	III	CHD
4	62	F	20	73	78	IV	DCM
5	58	F	29	65	70	III	DCM

*LVEF, left ventricular ejection fraction; LVESD, left ventricular end systolic dimension; LVEDD, left ventricular end diastolic dimension; CHD, coronary heart disease; DCM, dilated cardiomyopathy.*

### Implantation Data and Follow-Up

The left ventricular leads used for this technique included ST. JUDE medical 1458 (4 patients) and Medtronic 4195 (1 patient). At implantation, the threshold, impedance, and R wave of the left ventricular lead were 1.48 ± 0.18 V, 812.6 ± 95.7 ohms, and 11.6 ± 1.8 mV, respectively. At 12 months post-operation, the threshold, impedance, and R wave of the left ventricular lead were 1.15 ± 0.23 V, 850.6 ± 174.4 ohms, and 10.7 ± 0.7 mV, respectively. The pacing and sensing parameters were satisfactory in all patients at implantation and 12 months post-operatively (Table 2).

**TABLE 2:** Pacing and sensing parameters for left ventricular lead with the loop technique.

Left ventricular lead	Patient no. 1	Patient no. 2	Patient no. 3	Patient no. 4	Patient no. 5
Manufacturer/type of lead/length of lead/introducer size	ST. JUDE medical/1458/86 cm/7 Fr	ST. JUDE medical/1458/86 cm/7 Fr	ST. JUDE medical/1458/86 cm/7 Fr	Medtronic/4195/78 cm/8 Fr	ST. JUDE medical/1458/86 cm/7 Fr
**At implantation**					
Threshold (V)	1.3	1.6	1.5	1.3	1.7
Impedance (ohms)	690.0	750.0	910.0	903.0	810.0
R wave (mV)	12.0	10.4	14.0	10.6	11.0
**At 12 months post-operation**					
Threshold (V)	0.9	1.4	1.0	1.1	1.4
Impedance (ohms)	830.0	560.0	975.0	988.0	900.0
R wave (mV)	9.6	11.5	10.2	11.0	10.1

Table 3 shows the left ventricular structure and function values at the 12-month follow-up. The LVEF, LVESD, and LVEDD were 33.4 ± 4.7%, 51.6 ± 8.7 mm, and 61.8 ± 8.6 mm, respectively. Compared with pre-operative values, the 12-month post-operative values were significantly increased for the LVEF and reduced for the LVESD and LVEDD (*P* < 0.05). The LVEF of those 5 patients increased by more than 15%. Therefore, CRT significantly improved the left ventricular structure and function of these 5 patients.

**TABLE 3:** Patient characteristics at the 12-month follow-up with the loop technique.

Patient no.	LVEF (%)	LVESD (mm)	LVEDD (mm)	NYHA post-operative
1	36	47	54	II
2	39	46	58	II
3	29	60	70	II
4	28	62	72	II
5	35	43	55	II

*LVEF, left ventricular ejection fraction; LVESD, left ventricular end systolic dimension; LVEDD, left ventricular end diastolic dimension.*

**FIGURE 3:**
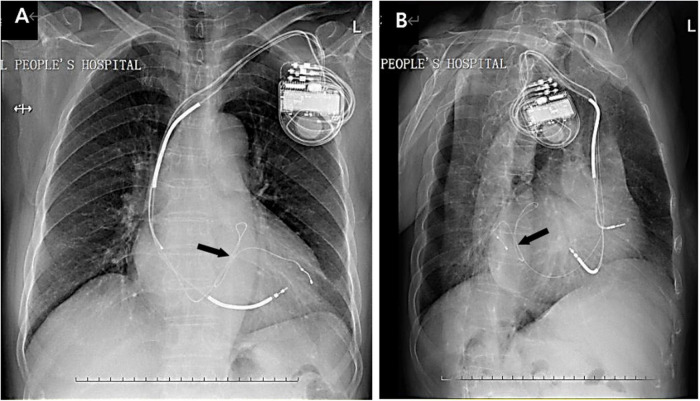
Chest X-ray images from anteroposterior projection **(A)** and lateral projection **(B)** show the left ventricular lead implanted through the coronary sinus by the loop technique at the 12-month follow-up. The arrows show the loop in the location of the coronary vessel.

At hospital pre-discharge and at the 1-, 3-, 6-, and 12-month follow-ups, no patient had died, there were no CRT infections, and no lead dislocation were observed. Figure 3 shows a chest X-ray image of the left ventricular lead implanted by the loop technique at the 12-month follow-up.

## Discussion

Cardiac resynchronization therapy has become a valuable non-drug treatment for refractory symptomatic heart failure, severe left ventricular systolic dysfunction, and ventricular dyssynchrony in patients with inadequate response to optimal medical therapies (12). CRT has the potential to improve heart failure symptoms, reduce hospital admissions, and improve the survival and functional capacity. The mechanisms of benefits from CRT include improved left ventricular remodeling resulting in reduced left ventricular size and increased ejection fraction, as well as mitigation of mitral regurgitation (5, 13). With the advancement of retrograde coronary sinus cannulation technology, delivery systems and dedicated left ventricular lead technology, the fully transvenous route has become the first-line approach for CRT (14). Percutaneous placement of the right leads is feasible in almost all cases. However, CRT involves implanting pacing leads into the left ventricles through the coronary sinus, which has a significant incidence of lead dislocation at implantation and during follow-up (8). Intraoperative lead dislocation may also lead to suboptimal left ventricular lead location, which may reduce the hemodynamic improvement effect of resynchronization therapy (15, 16). According to current experience, the inability to achieve a stable left ventricular lead position in the target vessel at implantation may occur in at least some patients. To achieve CRT, effective methods are needed for patients with repeated intraoperative left ventricular lead dislocations through the coronary sinus (17).

The loop technique used in this study provides a safe and effective solution for patients with repeated intraoperative left ventricular lead dislocation. In five patients with repeated intraoperative dislocation during transvenous left ventricular lead implantation, this loop technique was successfully used to stabilize the lead in its final position. In order to facilitate the formation of the loop and fix lead, long lateral/posterolateral vein and a reasonable amount of backup by a guiding catheter were needed. Intra-operative and post-operative pacing and sensing parameters at the 12-month follow-up were satisfactory in all patients. During the 12-month follow-up, no patient had died, there were no CRT infections, and no lead dislocations were observed. CRT improved the left ventricular structure and function, the functional status, and the hemodynamic status.

De Cock et al. used a retained guidewire technique for repetitive intraoperative transvenous left ventricular lead dislocations (15). Although this is a fast and simple solution, the presence of the guidewire inside the lead may cause damage to the inner coil and eventually lead to wire fracture due to prolonged friction. At the same time, modification of pulse propagation may be affected by the specific materials of guidewires from various manufacturers. Atrial transseptal left ventricular endocardial lead placement has been proposed and appears to be feasible in a cohort of 10 patients (18). Left ventricular endocardial pacing *via* interventricular septal puncture may also be effective and durable, with significant but potentially acceptable risks in a cohort of 15 patients (19). Leadless left ventricular endocardial pacing electrodes have also been used clinically (20).

In case of failure of transvenous left ventricular lead placement (including coronary sinus anatomical abnormalities, venous obstruction, unintended left phrenic nerve stimulation, transvenous lead dislocation that cannot be resolved), surgery can be considered and direct surgical epicardial left ventricular lead placement may provide the potential to pace the optimal target site. Although both the median sternotomy and left thoracotomy are available, minimally invasive approaches reduces the risk of surgery and the incidence of complications. Ceresa et al. reported their experience with CRT through a left minithoracotomy after failed transvenous approach (21). The MyoPore sutureless myocardial pacing lead can be implanted through a left minithoracotomy (∼5 cm) under selective right lung ventilation. The operation time was 38.5 ± 3.0 min, and there were no operative complications or early death. After a mean follow-up of 10.7 months, there were no cases of death, dislodgement, or loss of capture of the lead (21). Transapical endocardial left ventricular lead placement to the lateral free wall has been described in one case report and appears to be feasible, especially in cases of extensive pericardial adhesions from previous cardiac surgery (22).

In recent years, improvements in left ventricular leads and delivery systems have made CRT more accessible to patients with heart failure. In clinical practice, the standard first-line approach is the transvenous epicardial left ventricular lead placement through coronary sinus. The surgical access is most frequently used as a second choice. At present, there is a lack of data on the safety and effectiveness of some new techniques, but these methods may provide an alternative to left ventricular lead dislocations.

## Limitations

However, it must be acknowledged that there are some limitations to our findings. Although no adverse outcomes of the lead were observed during the short-term follow-up, it is necessary to extend the follow-up time. This loop technique was used in only 5 patients in this article. Therefore, additional studies with more patients are needed to explore the clinical value of this technique. However, we believe that until these results are available, this loop technique can be used as a means for repetitive left ventricular lead intraoperative dislocation through the coronary sinus if the standard implantation procedure fails.

## Conclusion

In summary, this is the first report that shows the feasibility of repetitive intraoperative dislocation of the left ventricular lead through the coronary sinus using the loop technique. We have demonstrated that this loop technique is safe and effective, and it can be considered for repeated intraoperative dislocation during transvenous left ventricular lead implantation through the coronary sinus of a CRT device.

## Data Availability Statement

The raw data supporting the conclusions of this article will be made available by the authors, without undue reservation.

## Ethics Statement

The studies involving human participants were reviewed and approved by Medical Ethical Review Board of Shaanxi Provincial People’s Hospital. The patients/participants provided their written informed consent to participate in this study.

## Author Contributions

All authors contributed to the patient care, diagnosis, treatment, and the writing of this manuscript and approved the submitted version.

## Conflict of Interest

The authors declare that the research was conducted in the absence of any commercial or financial relationships that could be construed as a potential conflict of interest.

## Publisher’s Note

All claims expressed in this article are solely those of the authors and do not necessarily represent those of their affiliated organizations, or those of the publisher, the editors and the reviewers. Any product that may be evaluated in this article, or claim that may be made by its manufacturer, is not guaranteed or endorsed by the publisher.
